# Multimatrix Detection and Quantification of the Advanced Glycation End Products Precursor Fructoselysine via UHPLC-HRMS/MS

**DOI:** 10.3390/metabo16010078

**Published:** 2026-01-16

**Authors:** Simona Fenizia, Marcello Manfredi, Valentina Antoniotti, Sabrina Tini, Jessica Baima, Flavia Prodam, Elettra Barberis

**Affiliations:** 1 Department of Translational Medicine (DIMET), University of Piemonte Orientale, I-28100 Novara, Italy; simona.fenizia@uniupo.it; 2 Center for Translational Research on Allergic and Autoimmune Diseases (CAAD), University of Piemonte Orientale, I-28100 Novara, Italy; elettra.barberis@uniupo.it; 3 Institute for Molecular and Translational Cardiology (IMTC), IRCCS Policlinico San Donato, San Donato Milanese, I-20097 Milan, Italy; 4 Department of Health Sciences, University of Piemonte Orientale, I-28100 Novara, Italy; valentina.antoniotti@uniupo.it (V.A.); sabrina.tini@uniupo.it (S.T.); jessica.baima@uniupo.it (J.B.); flavia.prodam@med.uniupo.it (F.P.); 5 Department of Sciences and Technological Innovation, University of Piemonte Orientale, I-15121 Alessandria, Italy

**Keywords:** AGEs, fructoselysine, liquid chromatography–tandem mass spectrometry, multimatrix quantification, pre-diabetic, aging

## Abstract

**Background:** Advanced glycation end products (AGEs) play a pivotal role in various human pathologies, including aging and metabolic diseases, and their formation may have significant physiological consequences for human health. Fructoselysine (FL) is an intermediate in the formation of AGEs, and its accumulation has been associated with detrimental health effects. Although several chromatographic methods have been developed for AGEs detection and quantification, no mass spectrometry-based approach has previously been established to quantify FL in different human biological matrices. **Methods:** In this study, we present a novel UHPLC-HRMS/MS method for the identification and quantification of this compound in various biological matrices, including plasma, feces, and urine. **Results:** The method demonstrates excellent linearity, accuracy, and precision, with limit of detection (LOD) of 0.02 µM and limit of quantification (LOQ) of 0.06 µM. Recovery rates ranged from 95% to 109% and intra- and inter-day relative standard deviations (RSDs) were below 10%, indicating robust analytical performance. The validated method was successfully applied to quantify FL in plasma, feces, and urine samples from healthy individuals. Additionally, given the known association between AGEs and diabetes, we analyzed a small cohort of prediabetic patients and observed elevated circulating levels of FL compared to healthy controls. **Conclusions:** This study introduces a sensitive and reliable method for the specific detection and quantification of FL in biological samples and provides new insights into early molecular changes associated with prediabetic condition to improve early diagnosis in aging related diseases.

## 1. Introduction

Fructoselysine (FL) is a glyco-amino acid formed through the Maillard reaction, where the ε-amino group of lysine reacts with a reducing sugar, namely glucose. The Maillard reaction is a non-enzymatic organic reaction, also known as glycation, that spontaneously occurs in biological systems (endogenous glycation) and in processed food (exogenous glycation) [1,2,3,4]. The reaction was discovered in 1912 by Louis Camille Maillard: the first stage of this nucleophilic addition occurs between free amino groups of specific proteins and sugars [5]. This step leads to the formation of reversible Schiff bases, which rearrange to more stable Amadori products. Once formed, Amadori products are further converted into reactive carbonyl species, including dicarbonyl compounds, such as glyoxal and methylglyoxal, which irreversibly bind to the side chains of proteins, leading to protein crosslinks and formation of stable advanced glycation end products (AGEs) [1,2,6,7].

The occurrence of the Maillard reaction in the human body and the endogenous AGEs formation was demonstrated for the first time in 1968, when Rahbar and colleagues showed that the exposure of hemoglobin to blood glucose leads to the formation of glycated hemoglobin, whose levels are altered in diabetic patients [3,8,9,10]. This discovery shed a new light on the impact of the Maillard reaction and the formation of endogenous AGEs on human health, including aging [11], hyperglycemia, and oxidative stress conditions, but also chronic degenerative diseases, like diabetes, cardiovascular diseases, and some types of cancer [12,13,14,15].

Recently, a broad variety of the Maillard reaction products (e.g., furosine, carboxymethyllysine, and pyrraline) have been investigated as markers for nutritional evaluation of heat-treated food [14]. Moreover, the consequences of their accumulation on both food quality and human health, as well as methods to inhibit the Maillard reaction, have been extensively studied [14]. In addition to the Maillard reaction, endogenous AGEs and their intermediates can be generated within the human body through four other metabolic pathways, namely the polyol pathway, glycolysis, glucose autoxidation, and lipid peroxidation [3,9,10,15]. The endogenous AGEs production is rather slow and prolonged compared to the rapid formation of the exogenous ones obtained during food preparation, mainly due to a lower temperature in the human body [10].

Whether they are exogenously or endogenously derived, the variety of AGEs’ formation mechanisms and their different sources and precursors make these molecules very diverse and heterogeneous, with many structures yet to be deciphered.

Many studies are reported in the literature, where some representative AGEs have been measured through colorimetric and fluorometric techniques and immunochemical methods, such as Western blotting and ELISA [12], and bioanalytical methods, including liquid chromatography coupled to mass spectroscopy [16]. Poojary et al. developed an analytical LC-MS/MS-based method for the comprehensive analysis and quantification of major AGEs found in processed foods as well as in biological samples [17]. Several methods for quantifying some AGEs in biological fluids or tissues have been developed; however, despite the high interest and concern regarding the physiological consequences that accumulation of AGEs intermediates may bring to human health, only a few studies have focused on the Maillard reaction product fructoselysine and its determination and quantification in biological matrices [18,19,20,21,22]. Moreover, a specific and standardized method to measure these compounds is required to enhance the detection and applications of AGEs’ evaluation for clinical purposes.

In this research, a new method to accurately identify and quantify fructoselysine was established. Using ultra-high-performance liquid chromatography coupled with high resolution tandem mass spectrometry (UHPLC-HRMS/MS), the study leverages the advanced sensitivity and specificity of this analytical technique. This approach ensures precise and reliable detection and quantification of this compound. To elucidate the involvement of FL in human health, the quantification of this metabolite was performed in three different biological matrices: plasma, stool, and urine. Then, the method was applied to investigate FL levels in prediabetic patients and healthy subjects.

## 2. Materials and Methods

### 2.1. Chemicals and Reagents

Fructoselysine (Fructosyl-lysine) standard was purchased from MedChemExpress (accessed on 6 November 2023, MCE, https://www.medchemexpress.com, Monmouth Junction, NJ, USA). Acetonitrile (ACN) and water (H_2_O), both HPLC-MS grade and suitable for UPLC/UHPLC instruments, were purchased from VWR International S.A.S. (Rosny-sous-Bois, France). Methanol (MeOH, UHPLC/MS grade) was purchased from Scharlab S.L. (Sentmenat, Barcelona, Spain). Formic acid (98–100% for analysis) was purchased from Merck KGaA (Darmstadt, Germany).

### 2.2. Preparation of Standard Stock Solutions and Spiked Matrices

First, 10 mg of the fructoselysine standard was dissolved in 1.621 mL of H_2_O, resulting in an initial concentration of 20 mM. This solution was further diluted to 10 µM, which was used as a working stock solution. Afterward, suitable dilutions (i.e., 0.05–0.5–5 µM) in H_2_O were prepared from the stock solution to obtain the working solutions used for matrix spiking and calibration curves. All solutions were stored at −20 °C.

### 2.3. Biological Samples and Sample Preparation

Plasma, urine, and fecal samples from healthy volunteers and prediabetic patients were collected and kept at −80 °C until analysis. Samples were collected at the Maggiore Hospital of Novara. The protocol was conducted following the Declaration of Helsinki (World Medical Association Declaration of Helsinki 2013), approved by the Ethical Committee of “Maggiore della Carità” Hospital of Novara (protocol CE 167/19) and registered on Clinicaltrials.gov (NCT04495972). Informed consent has been obtained from patients before study evaluations and after careful explanations to each one. All patients provided written informed consent.

Extraction of FL from plasma and urine was performed by transferring 40 µL of each matrix into a 1.5 mL Eppendorf^®^ tube and adding 200 µL of cold MeOH and 100 µL of ACN. Samples were vortexed and kept at −20 °C for 2 h to enhance protein precipitation. After centrifugation (15 min, 4 °C, and 15,000 rpm), the supernatant was collected, dried in SpeedVac, and resuspended in 150 µL MeOH:H_2_O 1:1. Prior to UHPLC-MS/MS analysis, samples were centrifuged (5 min, 4 °C, 15,000 rpm) to remove any additional particle, and the entire volume (150 µL) was transferred to a glass HPLC vial with an insert. FL extraction from fecal samples was performed by adding 200 µL of H_2_O, 200 µL of MeOH, and 100 µL of ACN to 30 mg of feces. Samples were then vortexed, sonicated for 5 min, and homogenized (FastPrep-24™ 5G, MP Biomedicals, Solon, OH, USA) for 3.3 min (5 cycles of 40 s each, speed 6.0 m/s). Likewise, as with plasma and urine samples, centrifugation was performed (15 min, 4 °C, and 15,000 rpm), and the supernatant was collected, dried in SpeedVac (ScanSpeed 40, ScanLaf, Analytical Control De Mori (ACDM), Milan, Italy), and resuspended in 150 µL MeOH:H_2_O 1:1. Prior to UHPLC-MS/MS analysis, an additional centrifugation step (5 min, 4 °C, 15,000 rpm) was performed, and the supernatant was transferred to a glass HPLC vial with an insert and kept at −20 °C until the analysis.

### 2.4. Chromatographic Separation and Mass Spectrometric Conditions

Chromatographic separation of FL was achieved on an ACQUITY UPLC BEH Amide column (2.1 × 150 mm, 1.7 μm, Waters, Milford, MA, USA) using a UHPLC Vanquish system (Thermo Fischer Scientific, Milan, Italy) coupled to a Q-Exactive Plus Hybrid Quadrupole-Orbitrap mass spectrometer (Thermo Fisher Scientific, Milan, Italy). The analytical method is based on a previous publication [23], which has been optimized as outlined below. The UHPLC column was operated at 45 °C. A constant flow rate of 0.26 mL/min was applied, with a binary gradient system of high-purity water and 0.1% formic acid (solvent A) and acetonitrile with 0.1% formic acid (solvent B). The initial gradient (2% B) was held for 1 min (0 to 1 min), increased linearly to 98% B over 8 min (1 to 9 min), held at 98% for 3 min (9 to 12 min). The system returned to the initial gradient (2% B) in 0.1 min (12 to 12.1 min) and was held at these conditions for 2.9 min (12.1 to 15 min) to re-equilibrate the column, thus resulting in a total run time of 15 min. Injection volume was 3 µL. Thermo Xcalibur software (version 4.1.31.9, Thermo Fisher Scientific, Milan, Italy) was used to control the instrument, acquire, and process the data.

The eluted analytes were analyzed with the Q-Exactive Plus Hybrid Quadrupole-Orbitrap mass spectrometer (Thermo Fisher Scientific, Milan, Italy), equipped with a heated electrospray ionization source (HESI-II, Thermo Fisher Scientific, Milan, Italy), using the following parameters: capillary temperature, 320 °C; spray voltage, 3800 V; sheath gas flow, 40 arbitrary units; aux gas flow, 10 arbitrary units. Acquisition was performed in positive ion mode, and data were acquired in full scan mode (FS) (from 100 to 1000 *m*/*z*, at a resolution of 70,000 full width at half maximum (FWHM)) and in time-scheduled parallel reaction monitoring (PRM) events to enhance the ion signal intensity. The time schedule for the PRM mode was determined using the start and end times of the FL precursor ion in FS mode and setting the collision energy to 30 eV in that time range to retain at least 10% of the unfragmented precursor ion. PRM was performed in positive ion mode, at a resolving power of 17,500 FWHM, automatic gain control (AGC) target: 2e5, a maximum injection time of 100 ms, with an isolation window of 4.0 *m*/*z*. FL standard was injected at a concentration of 2.5 µM at the end of the chromatographic run. Quality control samples (QCs) were prepared by mixing the same volume of each matrix after their extraction and solvent resuspension, and injected, without further dilutions, at the end of the chromatographic run and were also used for the method validation, as reported below.

Identification of fructoselysine was carried out by addition of synthetic fructoselysine (MedChemExpress) within the sample and by comparing retention time and fragmentation patterns of the same sample before and after the addition of the synthetic molecule.

### 2.5. Peak Extraction and FL Quantification

Identification and quantification of the FL was carried out using Thermo Xcalibur Qual Browser software (version 4.1.31.9, Thermo Fisher Scientific). An external calibration curve of the standard was prepared by diluting the 10 µM stock solution to obtain an 8-point calibration curve (0.05–0.125–0.25–0.5–1.25–2.5–5.0–10 µM), followed by comparisons of the peak area of the standard product ions with the peak area of the analyte product ions. As a matrix blank, BSA diluted with PBS was used to simulate serum matrix, while for urine and fecal matrices, diluted samples were used. In FS mode analysis, the FL peak was identified through the monoisotopic mass of the protonated molecule [M + H]^+^ = 309.1660, and its retention time (RT) was compared to that of the analytical standard.

In PRM mode, an extracted ion chromatogram was created using the accurate masses of the most intense fragment ion found in the FL standard and the unfragmented precursor ion at a mass tolerance of 5 ppm, and their areas were used for quantification. The areas under the curves obtained in full scan and PRM mode were also analyzed for the recovery percentage.

The calibration curve (*n* = 3) for the area of the molecular ion (*m*/*z* 309) was y = 9674884.50x − 779442, with R^2^ = 0.9988, limit of detection (LOD) = 0.025 µM, and limit of quantification (LOQ) = 0.077 µM. The calibration curve (*n* = 3) for the area of the fructoselysine product ion (*m*/*z* 128) was y = 675297.78x + 188221, with R^2^ = 0.999, limit of detection (LOD) = 0.020 µM, and limit of quantification (LOQ) = 0.062 µM. The calibration curves of the molecular ion and the product ion were used for quantification of FL in biological samples (Section 2.7. Quantification in Biological Samples).

### 2.6. Method Validation

The quantitative PRM method was validated through the analysis of the linearity, limit of detection (LOD), limit of quantification (LOQ) (Table 1), precision and accuracy, and recovery from biological samples (Table 2). Prior these analyses, the system was checked for its suitability by checking instrument status (calibration, mass accuracy), chromatographic quality (retention time, resolution, peak shape), and signal stability (response, carryover) using calibration solutions.

The method’s linearity was evaluated on the basis of an 8-point calibration curve (0–0.05–0.125–0.25–0.5–1.25–2.5–5.0–10 µM) of the FL analytical standard, dissolved in H_2_O. Three blank samples, consisting of ultrapure H_2_O without the addition of the standard, were analyzed before starting the measurement to eliminate the risk of contamination and after the last run at the highest concentration to determine and evaluate carry-over effects.

LOD and LOQ values were calculated using the following formulas:

*LOD* = 3.3 × *s/slope of the calibration curve*; *LOQ* = 10 × *s/slope of the calibration curve*,

where s = standard deviation of the peak areas of least detectable concentration. The chromatograms of the LOQ and the ULOQ are reported in the Supplementary Figure S1.

The accuracy of the method, defined as the closeness of test results obtained by the method to the true value, was determined by preparing three different concentrations (0.5–5–10 µM) of FL analytical standard and performing all the steps of sample preparation. After UHPLC-HRMS/MS analysis, the FL concentrations were calculated using the formula of the calibration curve. The resulting values were compared with the theoretically expected concentrations and expressed as percent recovery, calculated using the formula [24,25]:

% *Recovery* = (*calculated concentration*/*theoretical concentration*) × 100

The precision of the method was determined as the relative standard deviation (RSD%) for intra- and inter-day repeated injections [24,25]. Solutions of the FL standard (2.5 µM) as well as QCs (*n* = 3), prepared by mixing the same volume of each matrix after their extraction and solvent resuspension, were injected, without further dilutions, and analyzed at different time intervals on the same day to evaluate the intraday precision. The inter-day precision was measured by analyzing the same 2.5 µM solution and the QCs on three different days (Table 3) [24,25]. During the first 24 h, samples were stored at 4 °C in the autosampler of the UHPLC-HRMS/MS, while long-term storage was conducted at −20 °C.

### 2.7. Quantification of Biological Samples

FL was detected in biological samples using the described PRM method, and its quantification was achieved by applying the equation derived from the calibration curve of the product ion *m*/*z* 128 and the calibration curve of the monoisotopic mass of the protonated molecule [M + H]^+^.

### 2.8. Matrix Effects

Matrix effects were evaluated to identify any suppression or enhancement of FL in the different matrices. This effect was calculated by comparing the slopes of the analytical curves obtained from solutions prepared in organic solvent and those in the blank extract and calculated via the following equation [24,25]:

*Matrix Effect* = [(*slope curve of the standard in post-spiked matrix*) − (*slope curve of the standard in solvent*)/*slope curve of the standard in solvent*] × 100

## 3. Results

In the present research, a mass spectrometry method for the quantification of FL in human plasma, urine, and feces was developed. For human health, fructoselysine levels are linked to chronic diseases, dietary intake of AGEs, and cooking and food processing methods; therefore, its quantification in biological matrices is invaluable for understanding glycation processes and their impact on human health. To the best of our knowledge, UHPLC-HRMS/MS-based identification and quantification of this molecule in three biological matrices have never been reported. To achieve this, a UHPLC system combined with an Orbitrap mass spectrometer was employed for the detection and quantification of fructoselysine in plasma, stool, and urine samples, following an already established chromatographic separation method, based on a full scan analysis [23], to which we added a PRM analysis to enhance the detection sensitivity of this molecule.

**Figure 1:**
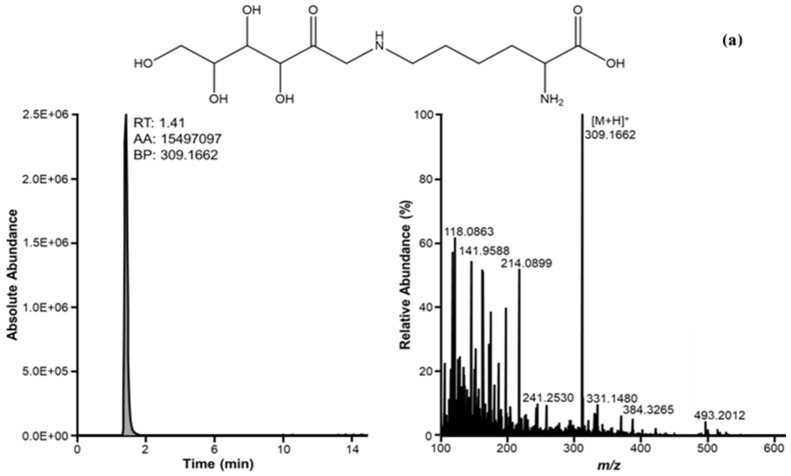

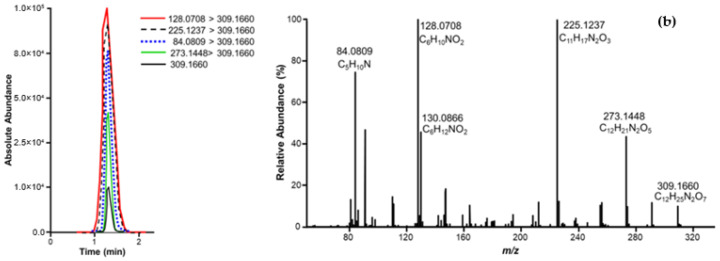
(**a**) The XIC (extracted ion chromatogram) of FL standard, analyzed in full scan mode (*m*/*z* = 309.1662; retention time: 1.41 min) (**left**) and the corresponding mass spectrum (**right**). (**b**) the overlapped XICs (**left**) of the precursor ion and four most abundant product ions obtained for FL in PRM mode (**right**). All chromatograms were acquired with a mass tolerance of 3 ppm.

The high resolution and high mass accuracy of the Q Exactive Plus Hybrid Quadrupole Orbitrap Mass Spectrometer enable it to achieve experimental mass accuracies less than 3 ppm for both precursor and fragment ions, and to determine the corresponding molecular formula. Figure 1 depicts the chromatogram and high-resolution mass spectrum of the FL, which shows a peak at RT = 1.42 min with [M + H]^+^ = 309.1660, corresponding to the molecular formula C_12_H_25_N_2_O_7_. The PRM-based analysis was initially performed on the FL standard solution in order to collect all the fragment ions from the FL precursor ion [M + H]^+^ = 309.1660; the same PRM approach was then applied on the biological samples, and the fragmentation patterns of the standard and the samples were compared. Three different collision energies (15, 30, and 40 eV) were tested; a collision energy of 30 eV was optimal for obtaining a complete fingerprint of the molecule, enhancing its detection and identification in biological samples.

HRMS/MS analysis of biological matrixes detected both the fragment ion *m*/*z* = 128.0709 and parent ion *m*/*z* = 309.1662 in all the biological samples used to develop the method. Co-elution and the matching of the MS/MS pattern confirmed the identity of FL and its occurrence in the analyzed matrixes [26]. Due to the high resolution of the Orbitrap mass analyzer, the base peak with *m*/*z* = 128.0709, which is the most abundant FL fragment, can be used for its quantification in biological matrixes. By performing external standard calibration curves and comparing the detector response to known concentrations of analyte with those obtained from biological samples having unknown FL amounts, we accurately identified and quantified this molecule in plasma, stool, and urine. This approach was selected over the internal standard addition because the corresponding labeled internal standard was not commercially available for this compound. The newly developed method was then tested for the bioanalytical method validation parameters required to ensure the correct quantification.

### 3.1. Method Validation Results

#### 3.1.1. Linearity, LOD, and LOQ

The linearity range was determined both in methanol and in matrices. Calibration curves of FL parent ion *m*/*z* 309 (y = 9674884.50x − 779442) and the product ion *m*/*z* 128 (y = 675297.78x + 188221) showed good linearities within the concentration range of 0.05–10 µM, as measured by the high values of coefficients of determination (R^2^) for both the ions, in particular R^2^ = 0.9988 for the parent ion *m*/*z* 309 and R^2^ = 0.9990 for the product ion *m*/*z* 128. Good linearity was also obtained in the three biological matrices, where, given the different physiological levels, distinct concentration ranges have been considered for each type of sample. Hence, plasma, urine, and fecal samples have been spiked, prior to the analysis, with different concentrations of FL standard (ranging from 0.25 to 2.5 µM for plasma, from 0.5 to 10 µM for urine, and from 0.125 to 10 µM for fecal samples), which resulted in linearity values (R^2^) related to the parent ion *m*/*z* 309 of 0.94, 0.97, and 0.97 for feces, urine, and plasma, respectively. These values were almost similar when the product ion *m*/*z* 128 was considered, resulting in R^2^ values of 0.93 for the fecal matrix, 0.98 for urine, and 0.98 for plasma. The slightly lower, but still accurate, linearities reported for the fecal matrix are mainly due to the high complexity and heterogeneity of this biological matrix. Fecal samples contain many more interfering substances such as bile acids, lipids, salts, and metabolites that make extraction and reproducibility challenging, leading to technical variation compared to other matrices, such as plasma and urine.

LOD and LOQ levels were calculated for both the parent ion *m*/*z* 309 and for the product ion *m*/*z* 128, using serial dilutions of the authentic FL standard, to confirm the sensitivity of the method. As shown in the table below (Table 1), in FS mode FL LOD was 0.025 µM and LOQ was 0.077 µM, while the LOD for the FL product ion *m*/*z* 128 was 0.020 µM and LOQ 0.062 µM, thus demonstrating the high sensitivity of the PRM method, which enhances data reliability by extracting the most appropriate fragmented ions.

**Table 1:** The developed method was evaluated for LOD, LOQ.

	FL Ions	R^2^	LOD μmol/L	LOQ μmol/L
**Product ion (*m*/*z*)**	128.0706	0.9990	0.020	0.062
**Parent ion (*m*/*z*)**	309.1660	0.9988	0.025	0.077

Chromatograms of the lowest (LOQ) and highest (ULOQ) concentrations of the FL standard used to build the calibration curve are reported in the Supplementary Materials.

#### 3.1.2. Accuracy and Precision

The accuracy of the method was determined by recovery studies. Hence, recoveries [%] obtained from the calibration curve and biological samples were calculated against the same concentration of the IS in matrix-free samples (Table 2). Each biological matrix was spiked with low, medium, and high concentrations of FL standard and extracted using the procedure described above. Recovery values range between 95% and 109%, which are acceptable values according to the analytical method development guidelines, indicating that the proposed method can be adopted for the analysis of this compound [25].

**Table 2:** Percentage of FL recovery calculated at low, medium, and high concentrations.

	Theoretical Concentration	FL Parent Ion Area (±RSD)	FL Product Ion Area (±RSD)	Amount Recovered (μmol/L)		Recovery (%)	
	(µmol/L)	*m*/*z* 309.1660	*m*/*z* 128.0706	*m*/*z* 309.1660	*m*/*z* 128.0706	*m*/*z* 309.1660	*m*/*z* 128.0706
	0.5	3.87 × 10^6^ (±7.08 × 10^5^)	3.87 × 10^5^ (±7.62 × 10^4^)	0.481	0.546	96%	109%
**FL** **Calibration**	5.0	4.51 × 10^7^ (±5.70 × 10^6^)	3.46 × 10^6^ (±4.45 × 10^5^)	4.745	5.094	95%	102%
	10.0	9.74 × 10^7^ (±1.53 × 10^7^)	6.76 × 10^6^ (±1.15 × 10^6^)	10.149	9.988	101%	100%
	0.5	3.17 × 10^6^	3.11 × 10^5^	0.508	0.528	102%	106%
**Samples**	5.0	3.94 × 10^7^	3.01 × 10^6^	4.900	5.297	98%	106%
	10.0	8.21 × 10^7^	5.61 × 10^6^	10.072	9.880	101%	99%

The precision of the method was assessed by intra- and inter-day precision (Table 3).

**Table 3:** Intra- and inter-day precision results.

Sample	Replicate	*m*/*z* = 128.0706 Measured Concentration	*m*/*z* = 309.1660 Measured Concentration	Intra-Day Variation (RSD%) *m*/*z* 128; *m*/*z* 309	Inter-Day Variation (RSD%) *m*/*z* 128; *m*/*z* 309
	R1	2.33	2.60		
**FL std 2.5 µmol/L**	R2	2.51	2.58	5.98; 1.17	6.01; 2.72
	R3	2.69	2.53		
	R1	9.02	5.87		
**QCs**	R2	8.48	5.85	3.1; 0.1	2.27; 2.62
	R3	9.10	5.86		

The RSD values for both intra- and inter-day precision were below 10%, indicating good precision.

#### 3.1.3. Carry-Over

No carry-over effect was identified when a solvent blank was injected three times after the highest calibration point on three consecutive days.

#### 3.1.4. Matrix Effect

Matrix effects were evaluated, and our results show that the full-scan analytical conditions required to perform the analysis resulted in negative matrix effect values. Hence, the different matrixes investigated in this work did not affect the quality of the results obtained. For the PRM peak *m*/*z* 128 in the plasma sample, a matrix effect of 3.57% was observed; however, the higher selectivity and sensitivity of the PRM method compared to the full scan make this approach reliable and feasible for research and clinical use.

### 3.2. FL Profiles in Biological Samples

The newly developed method was applied for the quantification of FL in plasma, feces, and urine from healthy subjects (*n* = 8) and prediabetic patients (*n* = 8). An initial determination of FL in healthy subjects was performed to identify its physiological levels.

Given the link between this molecule and the development of chronic and age-related diseases, as diabetes, the quantification of FL levels in plasma and feces collected from a cohort of prediabetic patients was performed. In healthy subjects, the quantification of FL in the same matrices and, additionally, in urine, was performed. In this population, the reported average of FL concentrations was 1.46 µM, 4.73 µM, and 9.86 µM in plasma, urine, and feces, respectively.

Interestingly, the levels of circulating FL were ten times higher (10.09 µM, *p*-value < 0.05) in prediabetic patients compared to healthy subjects, while fecal levels were pretty similar (7.29 µM). These results show the suitability of this method for the quantification of FL in biological matrices.

## 4. Discussion

In this study, we successfully developed and validated a novel UHPLC–HRMS/MS method for the identification and quantification of the advanced glycation end-products precursor FL in human plasma, urine, and feces. This compound is of increasing clinical relevance due to its association with dietary intake, food processing, and particularly with the pathogenesis of chronic and age-related diseases, including diabetes.

The method demonstrated excellent selectivity, enabling reliable identification and quantification of FL. The high-resolution performances of the Orbitrap mass analyzer and the use of PRM at an optimized collision energy of 30 eV ensured robust detection with very minimal matrix interferences.

Comprehensive validation showed strong linearity, sensitivity, and precision across all three biological matrices, with LODs as low as 0.020 µM and recovery values ranging from 95% to 109%.

The method also demonstrated minimal carry-over and manageable matrix effects, making it suitable for routine and translational research applications.

Application of this method to a small human cohort revealed quantifiable levels of FL in healthy individuals and prediabetic patients. Notably, plasma levels of FL were significantly elevated in prediabetic subjects compared to healthy controls, aligning with the hypothesized link between AGEs accumulation and early metabolic dysregulation. Fecal levels, however, remained relatively consistent across groups, suggesting tissue-specific or systemic differences in AGEs metabolism and excretion.

FL is an Amadori product mainly generated via the non-enzymatic Maillard reaction, a two-step reaction where the ε-amine group of lysine reacts with the reducing sugar glucose, forming an unstable intermediate (Schiff base) that rearranges into the more stable Amadori product FL [18]. Recent studies showed that gut microbiota can metabolize the exogenous FL into butyrate, a major representative short-chain fatty acid in the colon that plays important functions for intestinal health [18]. These findings suggest that gut microbial metabolism of fructoselysine may yield compounds involved in metabolization or bioremediation pathways, thus contributing to detoxification and benefits for human health [18]. Despite these potential beneficial aspects, FL is also a key reactive intermediate in the formation of potentially toxic advanced glycation end products. Studies showed that metal-catalyzed fragmentation of fructoselysine results in the production of reactive alpha-dicarbonyl species, such as glyoxal, which contribute to the formation of AGEs [18,27]. Moreover, after the exposure to hydroxyl radicals, FL generated carboxymethyllysine, one of the best-characterized AGEs that is causing widespread concern due to its use in various food products [28] and whose expression is associated with several age-related diseases and physiological processes, including cardiovascular disease, chronic inflammation, decreased brain function, and diabetes [29,30]. Indeed, FL-derived furosine was detected in the hair of diabetic patients, where it was significantly higher compared to healthy subjects and correlated with hemoglobin A1 values. Therefore, FL-derived furosine has been suggested as an indicator of plasma glucose levels and used for investigating diabetic complications [31].

Several experimental data demonstrated the association of AGEs with cardiovascular diseases and their higher incidence in individuals with diabetes, focusing on the measurement of two main AGEs derivatives, carboxymethyllysine, carboxyethyllysine, and pentosidine, in plasma samples of diabetic patients, demonstrating a positive correlation of these metabolites with fasting plasma glucose and HbA1c [32,33]. Moreover, the accumulation rate of AGEs is accelerated in diabetic conditions, especially in the bloodstream and body tissues (i.e., epithelial, connective, muscle, and nervous tissues) and organs, such as kidneys, retina, and atherosclerotic plaques, altering the vascular structure and function and leading to oxidative and inflammatory complications, cellular dysfunction, and multiorgan damage [34,35,36]. AGEs are also involved in chronic autoimmune connective disorders like systemic lupus erythematosus (SLE) and psoriasis. In SLE clinical studies have reported increased circulating AGEs alongside reduced soluble decoy receptor (sRAGE) levels, and more recent work supports associations between the AGE–sRAGE axis and disease activity and/or accrued damage, suggesting a role for glycoxidation-driven inflammation in lupus pathophysiology and vascular risk [37,38]. In psoriasis, AGEs are increased in serum and skin and correlate with disease severity [39,40]. Collectively, these findings position the AGE–RAGE pathway as a plausible immune–metabolic amplifier across chronic autoimmune connective and inflammatory skin disorders [40,41].

## 5. Conclusions

Our findings provide preliminary evidence supporting the clinical relevance of FL as potential biomarkers for early metabolic alterations and highlight the utility of the proposed analytical method in studying AGEs dynamics across multiple biological compartments. While the sample size was limited, the method’s performance demonstrates strong potential for future large-scale and longitudinal studies aimed at elucidating the role of glycation products in disease onset and progression. Further analysis in a larger, independent cohort of samples is required to confirm these results and assess their robustness and broader use.

The advantages and the accuracy of this novel methodology will accelerate the research on AGEs derivatives by providing more detailed and reliable data that will boost the understanding of the modes of action and the involvement of these molecules in human aging and related diseases. Overall, this method provides a comprehensive understanding of the AGEs’ derivative FL profile and offers a practical and effective option for use in clinical settings.

## Data Availability

The datasets generated during the current study are available from the corresponding author on reasonable request.

## Supplementary Materials

The following supporting information can be downloaded at: https://www.mdpi.com/article/10.3390/metabo16010078/s1, Figure S1: Lowest (LOQ) and highest (ULOQ) concentrations of the FL standard used to build the calibration curve.

## Abbreviations

The following abbreviations are used in this manuscript:

UHPLC-MS/MS	Ultra-High-Performance Liquid Chromatography tandem Mass Spectrometry
AGEs	Advanced Glycation End Products
sRAGE	Reduced Soluble Decoy Receptor
FL	Fructoselysine
LOD	Limit Of Detection
LOQ	Limit Of Quantification
RSD	Relative Standard Deviation
ACN	Acetonitrile
MeOH	Methanol
H_2_O	Water
PRM	Parallel Reaction Monitoring
FWHM	Full Width at Half Maximum
FS	Full Scan
RT	Retention Time

## References

[1] Cepas V., Collino M., Mayo J.C., Sainz R.M. (2020). Redox Signaling and Advanced Glycation End products (AGEs) in Diet-Related Diseases. Antioxidants.

[2] Chuyen N.V. (2006). Toxicity of the AGEs generated from the Maillard reaction: On the relationship of food-AGEs and biological-AGEs. Mol. Nutr. Food Res..

[3] Fournet M., Bonte F., Desmouliere A. (2018). Glycation Damage: A Possible Hub for Major Pathophysiological Disorders and Aging. Aging Dis..

[4] Spotti M.J., Loyeau P.A., Marangón A., Noir H., Rubiolo A.C., Carrara C.R. (2019). Influence of Maillard reaction extent on acid induced gels of whey proteins and dextrans. Food Hydrocoll..

[5] Maillard L.C. (1912). Reaction of amino acids on sugars: Formation of melanoidins by a systematic way. Comput. Rend. Acad. Sci..

[6] Ma H., Liu W., Frost L., Kirschenbaum L.J., Dain J.A., Seeram N.P. (2016). Glucitol-core containing gallotannins inhibit the formation of advanced glycation end-products mediated by their antioxidant potential. Food Funct..

[7] Delgado-Andrade C., Fogliano V. (2018). Dietary Advanced Glycosylation End-Products (dAGEs) and Melanoidins Formed through the Maillard Reaction: Physiological Consequences of their Intake. Annu. Rev. Food Sci. Technol..

[8] Rahbar S., Blumenfeld O., Ranney H.M. (1969). Studies of an unusual hemoglobin in patients with diabetes mellitus. Biochem. Biophys. Res. Commun..

[9] Chen J.H., Lin X., Bu C., Zhang X. (2018). Role of advanced glycation end products in mobility and considerations in possible dietary and nutritional intervention strategies. Nutr. Metab..

[10] Twarda-Clapa A., Olczak A., Bialkowska A.M., Koziołkiewicz M. (2022). Advanced Glycation End-Products (AGEs): Formation, Chemistry, Classification, Receptors, and Diseases Related to AGEs. Cells.

[11] Chaudhuri J., Bains Y., Guha S., Kahn A., Hall D., Bose N., Gugliucci A., Kapahi P. (2018). The Role of Advanced Glycation End Products in Aging and Metabolic Diseases: Bridging Association and Causality. Cell Metab..

[12] Perrone A., Giovino A., Benny J., Martinelli F. (2020). Advanced Glycation End Products (AGEs): Biochemistry, Signaling, Analytical Methods, and Epigenetic Effects. Oxid. Med. Cell Longev..

[13] Suzuki A., Yabu A., Nakamura H. (2022). Advanced glycation end products in musculoskeletal system and disorders. Methods.

[14] Erbersdobler H.F., Somoza V. (2007). Forty years of furosine—Forty years of using Maillard reaction products as indicators of the nutritional quality of foods. Mol. Nutr. Food Res..

[15] Ott C., Jacobs K., Haucke E., Navarrete Santos A., Grune T., Simm A. (2014). Role of advanced glycation end products in cellular signaling. Redox Biol..

[16] Corica D., Pepe G., Currò M., Aversa T., Tropeano A., Ientile R., Wasniewska M. (2022). Methods to investigate advanced glycation end-product and their application in clinical practice. Methods.

[17] Poojary M.M., Zhang W., Greco I., De Gobba C., Olsen K., Lund M.N. (2020). Liquid chromatography quadrupole-Orbitrap mass spectrometry for the simultaneous analysis of advanced glycation end products and protein-derived cross-links in food and biological matrices. J. Chromatogr. A.

[18] van Dongen K.C.W., van der Zande M., Bruyneel B., Vervoort J.J.M., Rietjens I.M.C.M., Belzer C., Beekmann K. (2021). An in vitro model for microbial fructoselysine degradation shows substantial interindividual differences in metabolic capacities of human fecal slurries. Toxicol. In Vitro.

[19] van Dongen K.C.W., Belzer C., Bakker W., Rietjens I.M.C.M., Beekmann K. (2022). Inter- and Intraindividual Differences in the Capacity of the Human Intestinal Microbiome in Fecal Slurries to Metabolize Fructoselysine and Carboxymethyllysine. J. Agric. Food Chem..

[20] van Dongen K.C.W., Ioannou A., Wesseling S., Beekmann K., Belzer C. (2022). Differences in gut microbial fructoselysine degradation activity between breast-fed and formula-fed infants. FEMS Microbiol. Ecol..

[21] van Dongen K.C.W., Kappetein L., Miro Estruch I., Belzer C., Beekmann K., Rietjens I.M. (2022). Differences in kinetics and dynamics of endogenous versus exogenous advanced glycation end products (AGEs) and their precursors. Food Chem. Toxicol..

[22] van Dongen K.C.W., Linkens A.M.A., Wetzels S.M.W., Wouters K., Vanmierlo T., van de Waarenburg M.P.H., Scheijen J.L.J.M., de Vos W.M., Belzer C., Schalkwijk C.G. (2021). Dietary advanced glycation endproducts (AGEs) increase their concentration in plasma and tissues, result in inflammation and modulate gut microbial composition in mice; evidence for reversibility. Food Res. Int..

[23] Xiao Z., Zhang Z., Huang S., Lon J.R., Xie S. (2022). Metabolic Profiling of Serum for Osteoarthritis Biomarkers. Dis. Markers.

[24] ICH Q2(R2): Validation of Analytical Procedures. https://www.ema.europa.eu/en/ich-q2r2-validation-analytical-procedures-scientific-guideline.

[25] Chavan S., Desai D. (2022). Analytical method validation: A brief review. World J. Adv. Res. Rev..

[26] Sargent M. (2013). Guide to Achieving Reliable Quantitative LC-MS Measurements.

[27] Brings S., Fleming T., Freichel M., Muckenthaler M.U., Herzig S., Nawroth P.P. (2017). Dicarbonyls and Advanced Glycation End-Products in the Development of Diabetic Complications and Targets for Intervention. Int. J. Mol. Sci..

[28] Wu Y., Li Y., Zheng L., Wang P., Liu Y., Wu Y., Gong Z. (2020). The neurotoxicity of Nε-(carboxymethyl)lysine in food processing by a study based on animal and organotypic cell culture. Ecotoxicol. Environ. Saf..

[29] Wang D., Wang J., Liu X., Du K., Liu H., Yang X., Liu T., Liu Q., Wang M., Guo J. (2024). Quantifying carboxymethyl lysine and carboxyethyl lysine in human plasma: Clinical insights into aging research using liquid chromatography-tandem mass spectrometry. BMC Biotechnol..

[30] Boesten D.M.P.H.J., Elie A.G.I.M., Drittij-Reijnders M.-J., den Hartog G.J.M., Bast A. (2014). Effect of Nɛ-carboxymethyllysine on oxidative stress and the glutathione system in beta cells. Toxicol. Rep..

[31] Oimomi M., Nishimoto S., Kitamura Y., Matsumoto S., Hatanaka H., Ishikawa K., Baba S. (1985). Increased fructose-lysine of hair protein in diabetic patients. Klin. Wochenschr..

[32] Hanssen N.M., Beulens J.W., van Dieren S., Scheijen J.L., van der A Daphne L., Spijkerman A.M., van der Schouw Y.T., Stehouwer C.D., Schalkwijk C.G. (2015). Plasma advanced glycation end products are associated with incident cardiovascular events in individuals with type 2 diabetes: A case-cohort study with a median follow-up of 10 years (EPIC-NL). Diabetes.

[33] Rabbani N., Thornalley P.J. (2015). Hidden complexities in the measurement of fructosyl-lysine and advanced glycation end products for risk prediction of vascular complications of diabetes. Diabetes.

[34] Goh S.Y., Cooper M.E. (2008). The role of advanced glycation end products in progression and complications of diabetes. J. Clin. Endocrinol. Metab..

[35] Rungratanawanich W., Qu Y., Wang X., Essa M.M., Song B.J. (2021). Advanced glycation end products (AGEs) and other adducts in aging-related diseases and alcohol-mediated tissue injury. Exp. Mol. Med..

[36] Uribarri J., Cai W., Peppa M., Goodman S., Ferrucci L., Striker G., Vlassara H. (2007). Circulating glycotoxins and dietary advanced glycation endproducts: Two links to inflammatory response, oxidative stress, and aging. J. Gerontol. A Biol. Sci. Med. Sci..

[37] Nowak A., Przywara-Chowaniec B., Blachut D., Nowalany-Kozielska E., Tyrpień-Golder K. (2021). Advanced glycation end-products (AGEs) and their soluble receptor (sRAGE) in women suffering from systemic lupus erythematosus (SLE). Cells.

[38] Carrión-Barberà I., Triginer L., Tío L., Pérez-García C., Ribes A., Abad V., Pros A., Monfort J., Salman-Monte T.C. (2024). Serum advanced glycation end products and their soluble receptor as new biomarkers in systemic lupus erythematosus. Biomedicines.

[39] Papagrigoraki A., Del Giglio M., Cosma C., Maurelli M., Girolomoni G., Lapolla A. (2017). Advanced glycation end products are increased in the skin and blood of patients with severe psoriasis. Acta Derm. Venereol..

[40] Kang P., Chen J., Wang S., Zhang S., Li S., Guo S., Song P., Liu L., Wang G., Gao T. (2023). Advanced glycation end products-induced activation of keratinocytes: A mechanism underlying cutaneous immune response in psoriasis. J. Innate Immun..

[41] Radziszewski M., Galus R., Łuszczyński K., Winiarski S., Wąsowski D., Malejczyk J., Włodarski P., Ścieżyńska A. (2024). The RAGE pathway in skin pathology development: A comprehensive review of its role and therapeutic potential. Int. J. Mol. Sci..

