# Supplementary material for: Multimatrix Detection and Quantification of the Advanced Glycation End Products Precursor Fructoselysine via UHPLC-HRMS/MS
Source: Metabolites. 2026 Jan 16;16(1):78. doi: 10.3390/metabo16010078 (PMC12843781; doi:10.3390/metabo16010078)
Supplement: Supplementary file 1 [file metabolites-16-00078-s001.zip › metabolites-4056647-supplementary.pdf]

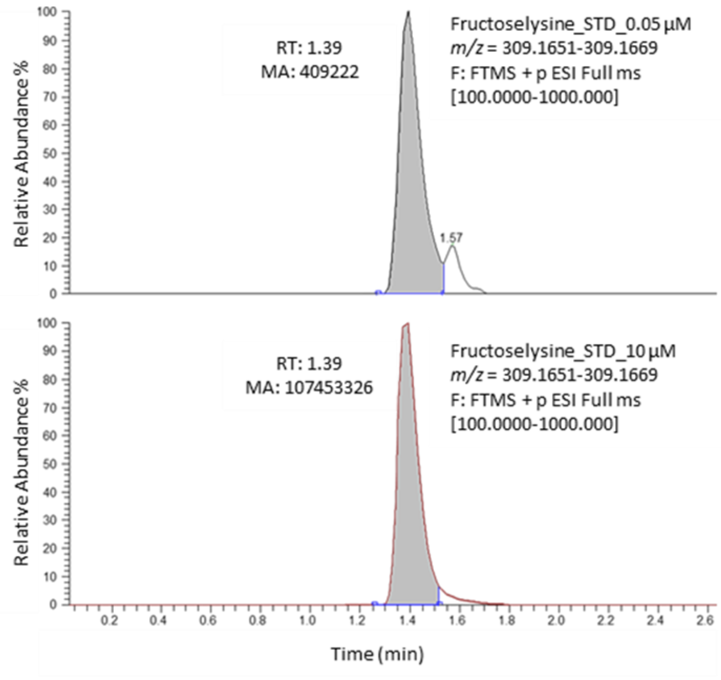

Figure S1. Lowest (LOQ) and highest (ULOQ) concentrations of the FL standard used to build the calibration curve. All chromatograms were acquired with a mass tolerance of 3 ppm.
